# CBCT image quality QA: Establishing a quantitative program

**DOI:** 10.1002/acm2.13062

**Published:** 2020-10-19

**Authors:** Sameer Taneja, David L. Barbee, Anthony J. Rea, Martha Malin

**Affiliations:** ^1^ Department of Radiation Oncology New York University Langone Medical Center New York NY USA

**Keywords:** cone‐beam computed tomography, image quality, institutional baselines, linear accelerator quality assurance

## Abstract

**Purpose:**

Routine quality assurance (QA) of cone‐beam computed tomography (CBCT) scans used for image‐guided radiotherapy is prescribed by the American Association of Physicists in Medicine Task Group (TG)‐142 report. For CBCT image quality, TG‐142 recommends using clinically established baseline values as QA tolerances. This work examined how image quality parameters vary both across machines of the same model and across different CBCT techniques. Additionally, this work investigated how image quality values are affected by imager recalibration and repeated exposures during routine QA.

**Methods:**

Cone‐beam computed tomography scans of the Catphan 604 phantom were taken on four TrueBeam® and one Edge™ linear accelerator using four manufacturer‐provided techniques. TG‐142 image quality parameters were calculated for each CBCT scan using SunCHECK Machine™. The variability of each parameter with machine and technique was evaluated using a two‐way ANOVA test on a dataset consisting of 200 CBCT scans. The impact of imager calibration on image quality parameters was examined for a subset of three machines using an unpaired Student’s *t*‐test. The effect of artifacts appearing on CBCTs taken in rapid succession was characterized and an approach to reduce their appearance was evaluated. Additionally, a set of baselines and tolerances for all image quality metrics was presented.

**Results:**

All imaging parameters except geometric distortion varied with technique (*P* < 0.05) and all imaging parameters except slice thickness varied with machine (*P* < 0.05). Imager calibration can change the expected value of all imaging parameters, though it does not consistently do so. While changes are statistically significant, they may not be clinically significant. Finally, rapid acquisition of CBCT scans can introduce image artifacts that degrade CBCT uniformity.

**Conclusions:**

This work characterized the variability of acquired CBCT data across machines and CBCT techniques along with the impact of imager calibration and rapid CBCT acquisition on image quality.

## 1 INTRODUCTION

Cone‐beam computed tomography (CBCT) scans are often performed using on‐board imaging (OBI) as part of image‐guided radiation therapy (IGRT) with linear accelerators.^1^ In contrast to diagnostic CT imaging, which often incorporates advanced image processing techniques to aid radiologists in diagnosis,^2^, ^3^, ^4^ linear accelerator‐based CBCT imaging typically uses filtered backprojection reconstruction techniques with the aim of providing an image with adequate soft tissue visualization that can be used to verify or improve patient alignment prior to treatment.^5^, ^6^ Cone‐beam computed tomography images can also be used to estimate the physical dose that patients receive during treatment,^7^ and are being explored for use in treatment planning^8^, ^9^ and adaptive radiotherapy.^10^ All of these uses require CBCT images to have adequate and consistent image quality: from adequate contrast for soft tissue visualization to consistent Hounsfield unit (HU) to attenuation coefficient mapping for dosimetric calculations.^11^ The increasing complexity and utilization of CBCT images require consistent CBCT performance, and thus a more stringent QA program.

Routine quality assurance (QA) of CBCT scanners tests for the consistency and adequacy of image quality metrics and helps ensure high‐quality, clinical CBCT scans. QA of CBCT systems on linear accelerators, including safety, mechanicals, and image quality, is outlined in the American Association of Physicists in Medicine (AAPM) Task Group (TG)‐142^12^ with further recommendations for CT‐based IGRT systems outlined in TG‐179.^13^ According to the guidelines of TG‐142, image quality metrics/parameters for CBCT images performed on a monthly basis include: geometric distortion, spatial resolution, contrast, HU constancy, uniformity, and noise.^12^ TG‐142 recommends a tolerance of “baseline” for all but geometrically measured metrics (Table 1) — indicating that image QA should evaluate for changes in image quality relative to what was measured during machine commissioning and are based on the individual institution’s data. In addition to TG‐142, linear accelerator vendors provide specifications for the image quality of their CBCT scans that should be met by scanners in clinical use (Table 1). Ideally, monthly CBCT QA would test for both changes in image quality and for meeting manufacturer specifications by the machine.

**Table 1:** Tolerances from TG‐142 and machine specifications for the Varian TrueBeam® for all cone‐beam computed tomography (CBCT) image quality metrics.

Parameter	TG −142	Varian TrueBeam®
Geometric distortion	<2 mm for non‐SRS/SBRT <1 mm for SRS/SBRT	N/A
Spatial resolution (full fan)	Baseline	≥6 lp/mm
Spatial resolution (half fan)	Baseline	≥4 lp/mm
HU uniformity	Baseline	±40 HU
Contrast	Baseline	N/A
Noise	Baseline	N/A
HU accuracy	Baseline	±50 HU

Establishing a monthly CBCT image quality QA protocol that aligns with TG‐142 requires determining a baseline value of each QA metric and the acceptable tolerances for measurements that are different from baseline. Image quality metrics will fluctuate between scans^14^ and the tolerance would ideally be set to accept these fluctuations while still being sensitive to actual changes in the image quality.^15^ Cone‐beam computed tomography QA procedures and expected CBCT image parameter fluctuations have been discussed by several authors. Yoo et al.^16^ used the OBI (On‐Board Imager®, Varian Medical Systems Inc.) on a Varian 2100 EX linac to evaluate CBCT image quality metrics on 4 months of multi‐institutional data using the Catphan® 504 phantom (Phantom laboratories, Greenwich NY). A single CBCT technique (125 kVp, 80 mA, 25 ms) was used and it was found that spatial linearity, scan slice geometry, contrast, and spatial resolution showed stable results over the timeframe. HU linearity was consistently within ±40 HU of the manufacturer's tolerances. Chang et al.^17^ investigated a 12‐month experience with the OBI on a single Novalis® treatment system in which image quality was measured with a Catphan® 504 phantom. Two CBCT techniques were investigated that included one full fan and one half‐fan configuration. It was found that image uniformity and noise agreed within a baseline value to within 1%. Stanley et al.^18^ investigated the stability of the OBI on a single Novalis® treatment system using a Catphan® 504 phantom and Pelvis and Head CBCT techniques over a 4‐month period. Standard deviations of the normalized results were used to report warning and action levels that ranged from 1% to 9% and 2% to 18%, respectively, for all image quality metrics for full‐fan CBCT modes and 1% to 17% and 2% to 34%, respectively, for half‐fan CBCT modes. According to the manufacturer of the Catphan® 604, 94 scans were completed using various scanners and protocols, and measurements of HU constancy yielded HU values that had ranges varying from 14 to 119 HU based on the material. It was also concluded that variability in kVp, slice thickness, object size, shape, and composition can change HU linearity.^14^

The studies listed above are specific to the machine types and imaging techniques that are investigated. There is limited published data on the fluctuations expected with newer TrueBeam® CBCT scans. Additionally, modern linear accelerators often have multiple CBCT techniques applicable for different anatomical sites and clinical situations and many institutions have multiple machines of the same model in clinical use. Previously published studies have not investigated how baselines and tolerances vary across machines and techniques. This information is needed to make an informed decision on whether baselines and tolerances should be technique‐dependent and to determine if departments with multiple machines will require machine‐specific or institution‐wide baselines. There are no published datasets and statistical analyses (mean, range, and confidence intervals) of measurements of image quality parameters across multiple imaging techniques on modern TrueBeam® accelerators. This information could be used by clinical physicists for comparison to their local data when establishing their own quantitative QA program with comprehensive baselines and tolerances. Finally, none of these studies has investigated the impact of imager recalibration on CBCT image quality QA results. As clinical physicists may recalibrate the kV imager at a set interval as part of machine maintenance, the information is important for determining if baselines need adjustment post‐calibration.

The purpose of this work was to address these limitations in the literature. This work investigated quantitative image quality results for five modern Varian machines (four TrueBeam® and one Edge®) and evaluated intra‐machine variability and inter‐machine variability. Baselines and tolerances were determined for each image quality metric based on technique, institutional data, and manufacturer recommendations. During data collection and analysis, it was found that there are additional, not previously reported, scenarios that may affect the measured image quality metrics. These included: (a) the impact of CBCT calibration (dark current, flood field, air normalization, and HU calibration) on image quality parameters and (b) the effect of monthly QA procedures on the image detector panel. The effect these scenarios have on monthly CBCT image quality results was quantified to enable other clinical users to incorporate the results into their monthly QA procedures.

## 2 MATERIALS AND METHODS

All CBCT acquisitions were performed using a Catphan® 604 phantom (Phantom Laboratory, Greenwich NY). This phantom is comparable to the Catphan® 504 phantom with some manufacturing differences that allow for additional test objects.^14^ All CBCTs were acquired using a series of Varian linear accelerators (Varian Medical Systems Inc., Palo Alto CA), including four TrueBeam® (called TB1, TB2, TB3, and TB4 in this work) and one Edge® (called Edge in this work) that were installed between 2014 and 2017. All machines were equipped with an amorphous silicon flat panel kV imaging detector. CBCTs were taken with the Varian TrueBeam® 2.7 MR3 software. Four Varian‐provided CBCT techniques were tested: Spotlight, Head, Pelvis, and Thorax (Table 2).

**Table 2:** List of cone‐beam computed tomography (CBCT) techniques acquired during monthly QA.

CBCT technique	Fan	Trajectory	kVp	mAs
Spotlight	Full	Full	125	750
Head	Full	Full	100	150
Pelvis	Full	Half	125	1080
Thorax	Full	Half	125	270

**Fig. 1:**
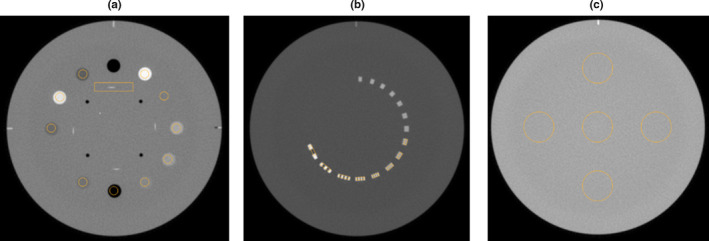
Registered cone‐beam computed tomography scan of the Catphan® 604 in SunCHECK Machine. Catphan modules shown in (a), (b), and (c) are used for analysis corresponding to parameters presented in Table 3.

Images were analyzed using SunCHECK Machine™ manufactured by Sun Nuclear Corporation (Melbourne, FL). Figure 1 shows the Catphan® 604 modules along with the regions of interest used for image analysis in the software. Table 3 shows a list of image quality parameters that are measured on a monthly basis as per the recommendations of the TG‐142 protocol.^12^

**Table 3:** Image quality parameters, units, and slice used for analysis.

Parameter	Units	Slice (Fig. 1)
Geometric distortion	mm	a
Spatial resolution	lp/mm	b
Uniformity	HU	c
Contrast	–	a
Noise	HU	a
Air HU constancy	HU	a
Teflon® HU constancy	HU	a
Delrin® HU constancy	HU	a
Acrylic HU constancy	HU	a
Polystyrene HU constancy	HU	a
Low density polyethylene (LDPE) HU constancy	HU	a
Polymethylpentene (PMP) HU constancy	HU	a
20% bone HU constancy	HU	a
50% bone HU constancy	HU	a
Slice thickness	mm	a

Calculation of image quality parameters in SunCHECK Machine™ follows recommendations from the Catphan® manufacturer.^14^ Geometric distortion is determined using four center holes that are 50 mm apart, and is calculated as the largest difference in absolute value between measurements of the center holes. Spatial resolution is measured by generating a modulation transfer function (MTF) by calculating the modulation for each spatial frequency region of interest (ROI) [Fig. 1(b)]. Uniformity is calculated using five ROIs, each approximately 2.9 cm in diameter, placed at the center and at the 3, 6, 9, and 12 o'clock positions around the periphery of the phantom [Fig. 1(c)]. The mean HU for each of the five ROIs is measured and the maximum difference between the mean value of each peripheral ROI and the central ROI is determined. The largest difference is reported as uniformity. Contrast, C, is calculated using Eq. (1):

(1) $$C=\frac{\left(A‐B\right)}{A}$$

where A is the mean gray scale value of the Teflon ROI, and B is the mean gray scale of the disc background ROI. Noise is calculated as the standard deviation of the background HU value. For determination of HU accuracy, SunCHECK Machine™ calculates the average HU, S_image_, in a given ROI [Fig. 1(a)] and compares the results to a reference HU S_reference_, which is set by the user. Each HU ROI is generated on a single image plane, is centered within the HU plug, and has a diameter of approximately 60% that of the HU plug. The maximum HU deviation, σ_HU_, determined by Eq. (2), is used.

(2) $$\sigma_{\text{HU}}=S_{\text{image}}‐S_{\text{reference}}$$

For determination of all image quality parameters, CBCTs were imported into SunCHECK Machine™, manually registered to the correct module, and ROIs were placed. All data were stored in SQL databases and analysis was completed using Excel (Microsoft Corporations, Redmond WA), and SPSS (IBM, Armonk NY).

### 2.A Machine and technique dependence

Two hundred CBCTs were taken across five linear accelerators and using four CBCT techniques over the course of 12 months. These CBCTs were taken as part of routine monthly QA procedures with additional CBCTs taken specifically for this work. Ten CBCTs were taken with each technique on each accelerator. No image calibrations were done on any of the linear accelerators during the period the CBCTs were taken. All monthly QA CBCTs taken during the year time frame were included in this analysis except for nine scans that did not follow institutional protocols for acquisition (ie, not aligned to isocenter or wrong CBCT technique was used). This set of CBCT acquisitions were used to determine the variability of each image quality parameter as a function of machine and technique. The difference in the mean values of each image quality parameter across machines and techniques was evaluated using two‐way ANOVA tests.

### 2.B CBCT calibration

The TrueBeam® 2.7 MR3 imaging system undergoes a calibration by the user before clinical use, after major service or part replacement, and at a user‐determined frequency thereafter. For CBCT techniques, the image calibration begins with a dark and flood field calibration of the dynamic gain kV mode (the kV mode used for CBCT scans). This is followed by a rotating air normalization calibration, which uses the same settings as the clinical CBCT technique, but the gantry is rotated through full clockwise and counter‐clockwise rotations. This scan is used to generate a fluence map of the radiation incident on a patient. Finally, the densitometry module of the Catphan® 604 is scanned to create an attenuation to HU curve. Individual calibration of any one component is possible without invalidating existing calibrations within the calibration set.

The impact of this imaging calibration on monthly imaging baselines was evaluated for three of the TrueBeam® machines. Image quality data collected before and after three of the machines underwent a new, full image calibration were analyzed to determine if the calibration altered the image quality data. SunCHECK Machine™ was used to analyze all data collected. Data from before the new image calibration were taken from routine monthly QA CBCTs and, for some of the machines, supplemented with additional CBCTs that were taken in between the monthly scans as part of other testing. Data from after the new image calibration were taken from routine monthly QA CBCTS and from additional CBCTs taken specifically to test the post‐calibration image quality. No monthly CBCT scans acquired with either the original or new calibration were excluded from the study. The number of CBCTs in each dataset varied with machine and technique, but ranged from 5 to 12.

### 2.C Artifacts

**Fig. 2:**
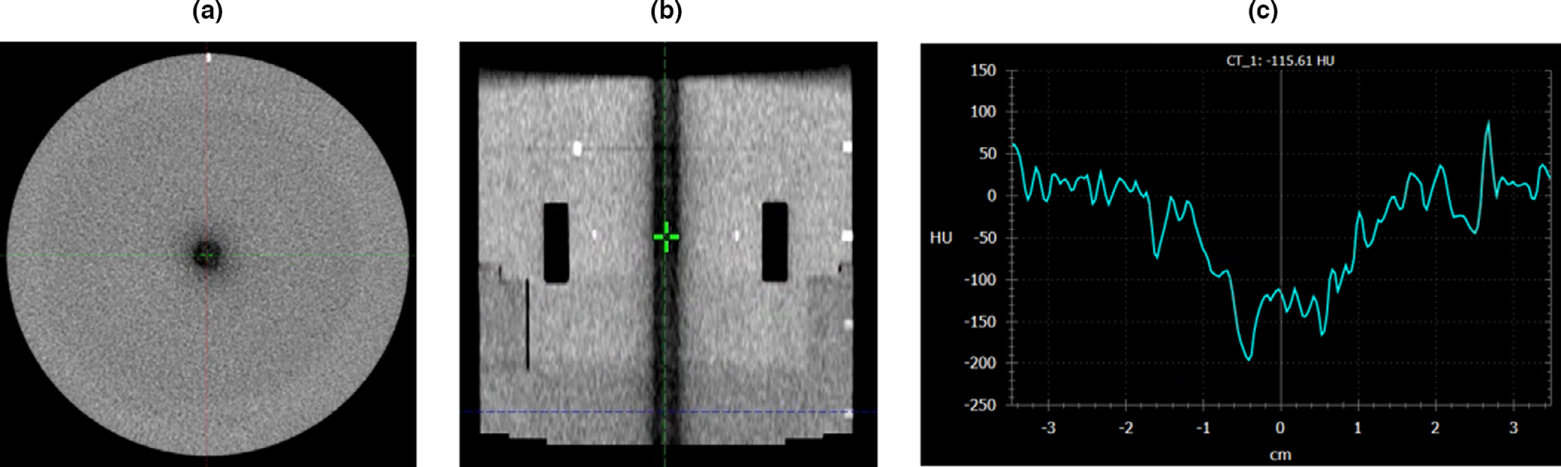
(a) Transverse and (b) sagittal planes of the module used for uniformity analysis of a cone‐beam computed tomography acquired using the Head technique and with the presence of a central dark artifact. (c) An HU line profile across the center of the transverse plane.

Initial data analysis showed that some CBCTs taken for routine machine QA exhibited a cylindrical artifact of lower HU values in the center of the phantom (Fig. 2). It was noticed that the artifact appeared when CBCTs were acquired in rapid succession, and presented on CBCT scans that were taken immediately after either a Pelvis or Spotlight scan (the two higher dose scans in this study) was taken. Scans taken using the Head and Pelvis techniques primarily showed the artifact. This work investigated what QA procedures induced the artifact and the effects of the artifact on image quality parameters, mainly uniformity.

### 2.D Baselines and tolerances

Baselines and tolerances were set for each image quality parameter based on institutional data. The dataset of CBCT measurements described in Section 2.A (N = 200) was used. The results from the ANOVA test of Section 2.A were used to identify the variability between machines and technique for each parameter, and the spread of the data was compared with the tolerance recommendations from the AAPM TG‐142 protocol and manufacturer specifications. This was used to identify an appropriate way to group data and set applicable baselines and tolerances that would account for the variability in measurements while also being able to identify significant changes in the image quality. These baselines and tolerances are presented as an example for other clinics that have multiple machines and test multiple techniques during monthly image quality QA.

## 3 RESULTS

### 3.A Machine and technique dependence

**Fig. 3:**
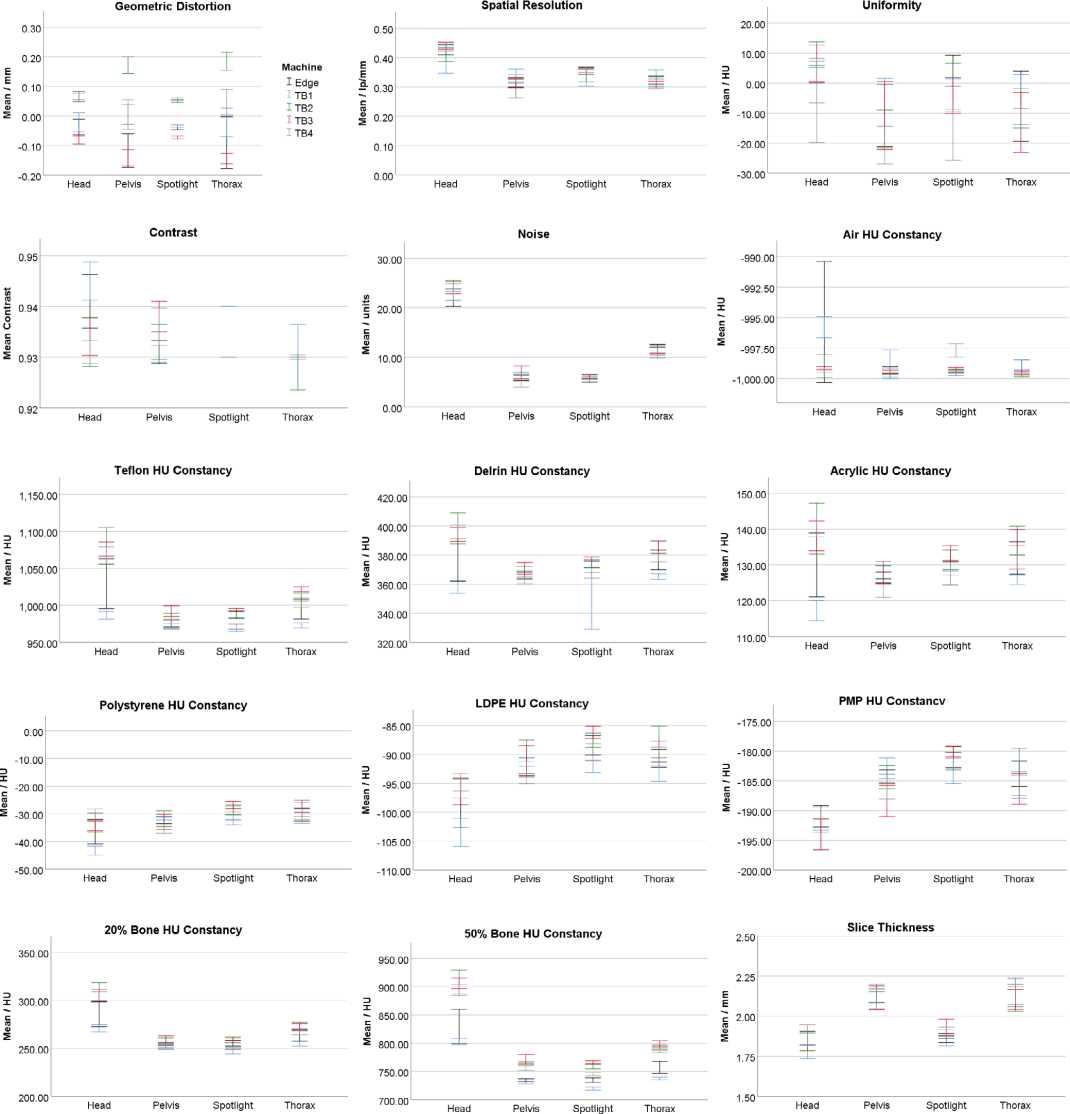
Institutional data (N = 200) for all image quality parameters separated by technique and machine and used for the two‐way ANOVA test. Error bars represent a 95% confidence interval.

Image quality parameters display a range of trends when evaluated graphically as a function of technique and machine (Fig. 3). As expected, parameters, such as noise, slice thickness, and spatial resolution, vary with technique while having relatively small spreads between machines for a given technique. Other parameters, such as the expected HU for Teflon, show a large, machine‐specific variation for some techniques (Head in the case of Teflon) along with a dependence of the expected value on the technique.

A two‐way ANOVA test (with machine and CBCT technique as factors) was performed in SPSS to test if image quality parameters were machine‐ and/or technique‐dependent. The two‐way ANOVA evaluated the hypothesis that the mean of each image parameter varied with the machine and technique used to measure it. Statistical significance was taken as having *P* < 0.05. The test also reports if there is an interaction between the machine and technique terms. When statistically significant, the interaction term indicates that the expected mean of a parameter is dependent on both the machine and the technique. Test statistics, degrees of freedom, and individual *P*‐values are reported in Supplemental Table S1. The two‐way ANOVA showed that all imaging parameters except geometric distortion varied with technique (*P* < 0.05) and all imaging parameters except slice thickness varied with machine (*P* < 0.05). The interaction term was significant for all image quality parameters except for uniformity, noise, and slice thickness.

### 3.B CBCT calibration

Cone‐beam computed tomographies were acquired pre‐ and post‐imager calibration with a total of 185 CBCTs in the dataset and a range from 5 to 12 CBCT image sets in each group (machine and technique). For each image quality parameter studied, a two‐tailed, unpaired Student’s t‐test was used to test the hypothesis that the mean value of that image quality parameter was different between data collected before and after the CBCT calibration procedure. Differences were considered significant if *P* < 0.05. The differences in the means between the pre‐ and post‐calibration data are shown in Table 4. Test statistics, degrees of freedom, and *p*‐values are reported in Tables S2–S5. Bold items in Table 4 are statistically significant. As shown in the table, the imager calibration procedure has the potential to change the expected value of all of the imaging parameters, though it will not always do so. While these changes are statistically significant, they may not be clinically significant. For example, the geometric distortion of the Thorax technique, measured on one machine, changed by 0.05 mm, but the TG‐142 tolerance for this test is 2 mm for machines that do not have stereotactic body radiation therapy (SBRT) capabilities.

**Table 4:** Difference in the mean between the pre‐calibration and post‐calibration data on three TrueBeam® machines (TB1, TB2, and TB3). Items in bold are statistically significant (*P* < 0.05).

Parameter	Head			Spotlight			Thorax			Pelvis		
	TB1	TB2	TB3	TB1	TB2	TB3	TB1	TB2	TB3	TB1	TB2	TB3
Geometric distortion (mm)	−0.03	0.00	−0.01	0.00	0.00	0.00	0.07	−0.01	**−0.05**	0.02	−0.02	−0.05
Spatial resolution (lp/mm)	0.00	0.01	0.03	0.00	**0.02**	0.01	**−0.04**	0.00	0.00	−0.04	0.03	0.01
Uniformity (HU)	**10.47**	−**30.73**	−7.39	**−12.95**	**−11.68**	**−14.04**	−10.10	**−12.16**	0.98	−14.61	**−17.26**	6.83
Contrast (−)	**−0.01**	0.01	0.00	−**0.01**	0.00	0.00	−0.01	0.00	0.00	0.00	0.00	0.00
Noise (HU)	1.73	**−2.02**	−0.41	0.00	−0.03	−0.57	**1.60**	0.48	0.30	−0.40	0.24	−0.97
Air HU constancy (HU)	**−3.20**	2.24	−0.16	**0.29**	**0.42**	0.04	**−0.47**	0.51	0.09	−1.23	**0.32**	0.06
Teflon HU constancy (HU)	**116.1**	−31.72	2.90	8.10	**−8.40**	−5.05	**42.62**	−9.40	−0.33	**14.19**	−4.75	−12.93
Delrin HU constancy (HU)	**50.62**	−14.53	2.11	4.57	**−4.53**	**−5.03**	**22.31**	−3.31	4.43	**7.84**	−1.09	−4.38
Acrylic HU constancy (HU)	**31.67**	−9.06	1.91	2.88	**−3.77**	**−6.16**	**17.63**	−3.23	2.30	4.89	−0.45	−4.91
Polystyrene HU constancy (HU)	**15.39**	−5.32	0.04	1.66	−2.03	**−4.66**	**10.14**	−1.05	0.70	3.22	−0.35	−3.98
LDPE HU constancy (HU)	**10.39**	−3.47	−1.26	1.78	**−2.91**	**−4.59**	**9.39**	−1.65	3.73	3.04	−0.35	−3.89
PMP HU constancy (HU)	4.05	−**3.98**	−0.18	2.10	−1.32	**−5.27**	**7.95**	−1.50	**6.17**	2.45	1.02	0.91
20% bone HU constancy (HU)	**39.82**	−13.41	6.18	1.51	**−6.45**	**−3.99**	**20.02**	−4.55	6.55	**7.44**	−1.41	−3.77
50% bone HU constancy (HU)	**93.39**	−25.59	−11.08	2.22	**−9.39**	**−15.70**	**34.12**	−6.30	−7.60	**10.07**	−3.13	−23.39
Slice thickness (mm)	**0.11**	0.01	0.00	0.04	0.02	−0.08	−0.07	0.03	0.06	−0.01	−0.02	0.04

**Fig. 4:**
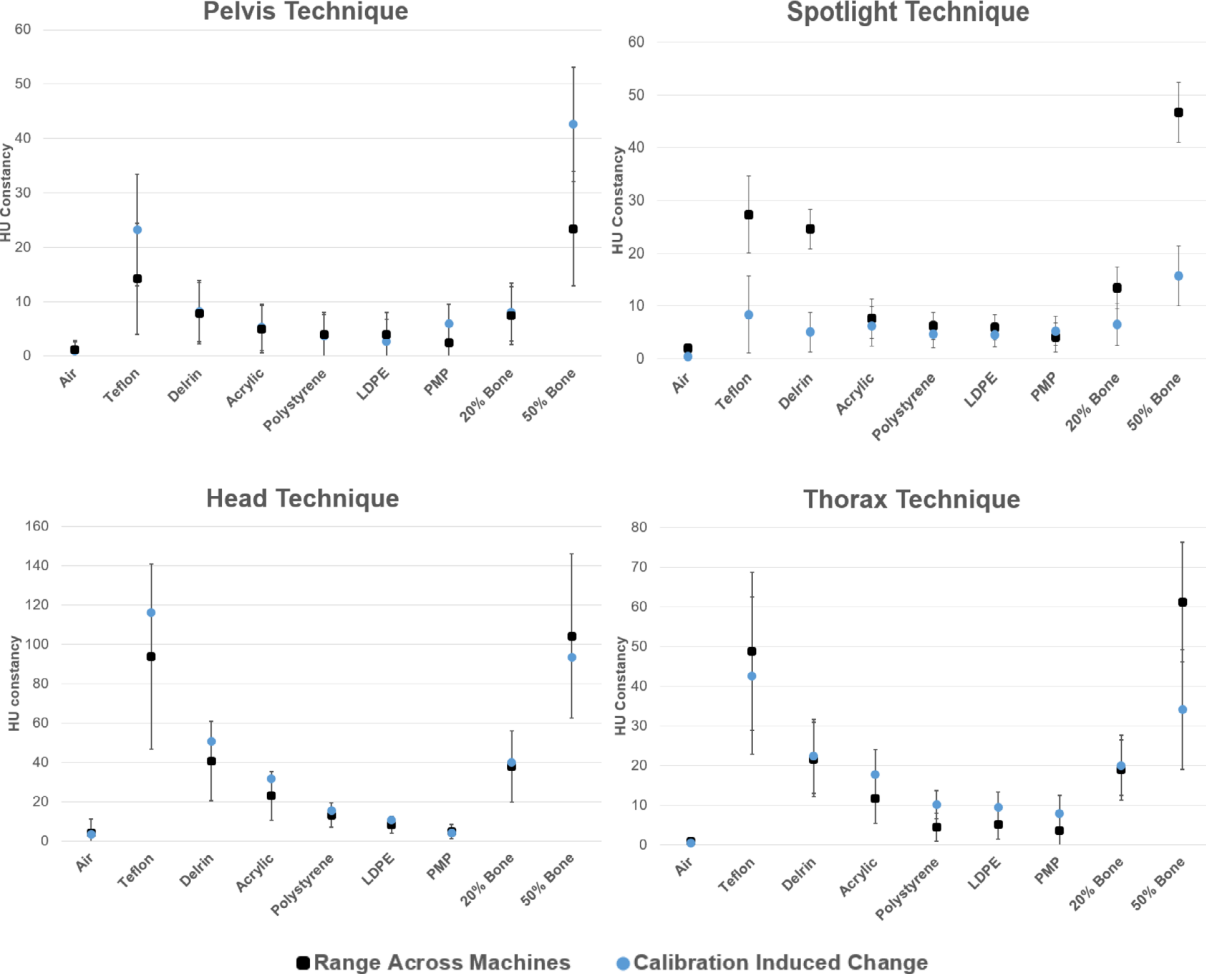
The maximum change in the average value of each HU plug caused by recalibration of the cone‐beam computed tomography imager plotted along with the range of expected values after calibration seen across all machines. Error bars are the largest, single machine standard deviation computed from post‐calibration data intra‐machine for each parameter.

The change in the expected value of the image quality parameters after calibration was compared to the machine‐to‐machine variability shown in Fig. 3. Figure 4 shows, for each technique, the range in the average value for a given image quality parameter across all machines. Also plotted is the largest change in the parameter due to recalibrating the CBCT technique reported in Table 4. With the exception of the Teflon, Delrin and 50% bone HU plugs for the Spotlight technique, the change in a given machine's expected HU value is within the range seen across machines. For the Teflon, Delrin and 50% bone HU plugs measured with the Spotlight technique, calibration‐induced changes to the expected value of the plug were less than the machine‐to‐machine variability.

### 3.C Artifacts

The artifact displayed in Fig. 2 was primarily produced in Head and Pelvis CBCTs that were completed immediately after a high‐dose scan and when the fan type was changed between the two scans. To characterize the impact of the artifact on the measurement of uniformity, pairs of scans were acquired using the following scanning protocols: (a) a Pelvis scan followed immediately by a Head scan and (b) a Spotlight scan followed immediately by a Pelvis scan. After each pair was acquired, there was a 10‐minute pause where no scans were taken. Following the pause, the last scan was repeated. This procedure was followed six times, three times for the Pelvis/Head/Head set of CBCTs and three times for the Spotlight/Pelvis/Pelvis set of CBCTs. At least, an hour was allowed to pass where no CBCT images were acquired on the machine before taking each set of three CBCT scans. All CBCT scans taken in this portion of the work were included in the analysis of this section of the work. The measured uniformity values from each scan are reported in Table 5. The average values of measured uniformity for the Pelvis and Head scans were −40.71 and −58.98 HU immediately after a high‐dose scan and −18.07 and −4.05 HU after a 10‐minute break, respectively. According to the Varian TrueBeam® manufacturer recommendations, the HU values for uniformity after a high‐dose scan are out of specifications (±40 HU), but the HU values post 10‐min interval are within. In addition, two consecutive Head scans were acquired with no time gap between scans and showed no presence of the artifact.

**Table 5:** Measurements of uniformity for cone‐beam computed tomographies (CBCTs) acquired immediately after a high‐dose scan and CBCTs acquired with at least a 10‐min interval between scans for the Head and Pelvis techniques.

Scan number	Post high‐dose scan		10‐min interval	
	Head	Pelvis	Head	Pelvis
1	−79.34	−30.68	−3.48	−17.02
2	−41.93	−43.05	−3.68	−16.31
3	−55.66	−48.39	7.29	−20.88
Average	−58.98	−40.71	−4.05	−18.07

**Fig. 5:**
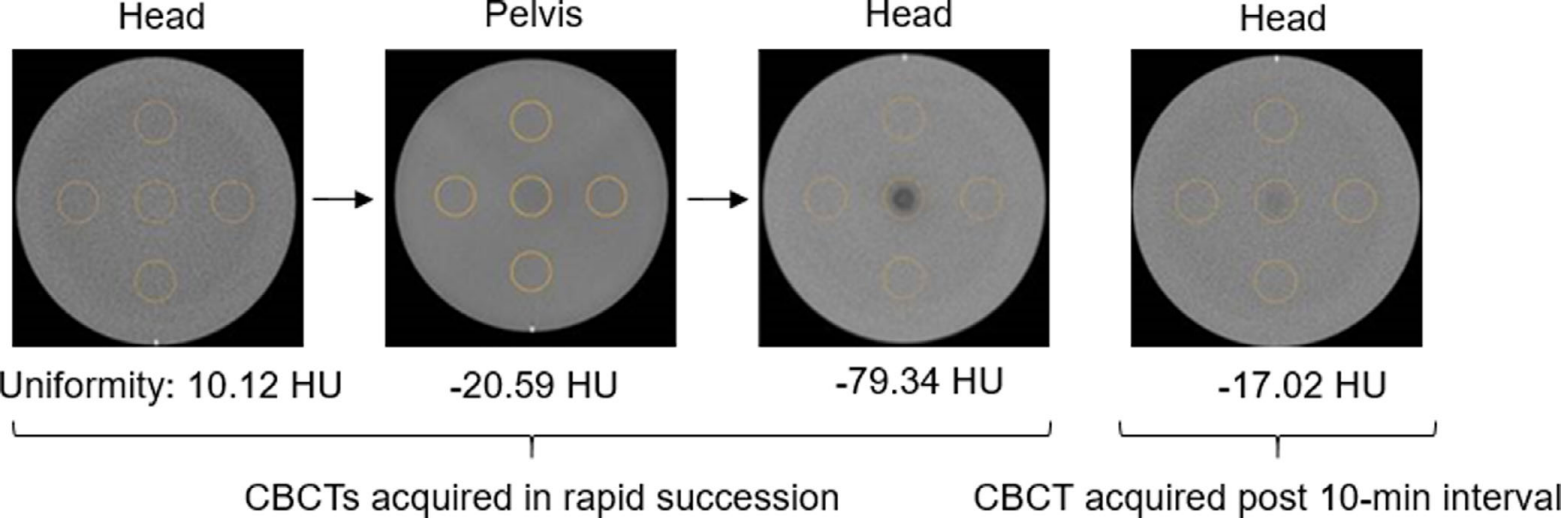
Catphan® uniformity module for cone‐beam computed tomographies (CBCTs) acquired in rapid succession and post 10‐min interval. The calculated uniformity value is presented for each CBCT.

As an example of the appearance and fading of the artifact, Fig. 5 shows the appearance of a central artifact in series of CBCTs taken in rapid succession. In SunCHECK Machine™, the central region of the image set is used for uniformity measurements, in which the HU values in the center and in the periphery of the module are measured and compared. A full‐fan Head CBCT performed immediately following a half‐fan Pelvis CBCT and a full‐fan Head CBCT displayed the artifact and caused the uniformity measurement to degrade from −20.59 HU on the Pelvis CBCT to −79.34 HU on the second Head CBCT. This is out of specification for a Varian TrueBeam® (specification of ±40 HU). A 10‐min break between CBCTs decreased the intensity of the artifact and the uniformity improved to −17.02 HU.

This artifact was not seen when a maximum of two CBCT scans were acquired in one session with the CBCTs having the same fan type (full or half fan). In‐house QA procedures were adjusted to follow this two scan, same fan‐type limit in order to minimize the presence of this artifact. Any CBCTs with this artifact were excluded from the analysis completed in other sections of this work if it was verified that the CBCTs with the artifact had been acquired rapidly after other CBCT scans.

### 3.D Baselines and tolerances

The CBCT dataset analyzed in Section 3.A was used to generate baselines and tolerances. With the exception of some high‐Z HU constancy tests that included the Teflon and 50% bone materials, the differences in image quality metrics between machines evaluated in Section 3.A fell within the guidelines of the AAPM TG‐142 protocol and the manufacturer’s specifications (Table 2) even though the differences were statistically significant. Additionally, as shown in Fig. 4, calibration‐introduced changes to the expected value for a given machine are consistent with the range of values found across machines. Baseline values for all image quality parameters except for geometric distortion and uniformity were set as the average of all measurements across machines for each CBCT technique (Table 5). Baselines for geometric distortion and uniformity were set to zero as this is the ideal value for these parameters and it was desired for tolerances to be relative to this value. Baseline values were defined to be technique‐specific because various CBCT acquisition settings, including kV, mAs, fan type, and trajectory, would be expected to affect image quality parameters, such as noise, slice thickness, and spatial resolution, as seen in Fig. 3.

The spread and standard deviation from Section 3.A were used to determine institutional tolerances, which are presented in Supplemental Table S6. Where specific tolerances were defined in either TG‐142 or the specifications of the Varian TrueBeam®, the institution adopted these tolerances. These included geometric distortion (1 mm), uniformity (40 HU), and HU constancy for non‐high‐Z material (50 HU). Two standard deviations (95% confidence interval) of the institutional data were used to set the results for spatial resolution, contrast, noise, and slice thickness based on the analysis found in Stanley et al.^18^ For HU constancy tests of high‐Z material, the tolerance was set to either 50 HU (manufacturer specification) or two standard deviations of the dataset, whichever was more lenient.

## 4 DISCUSSION

During the course of investigation, it was found that certain QA procedures affected the variability of CBCT image quality metrics. First, acquiring CBCTs in rapid succession during QA has the potential to produce a low‐HU artifact in the center of the reconstructed image. This artifact did not match, in intensity and shape, many previously characterized artifacts including cupping, streak, ring, aliasing, bar, halo, and speckle.^13^, ^19^, ^20^ A potential explanation for the cylindrical artifact is lag or ghosting of the detector panel, which can have a more variable effect on image quality and can result from a time‐dependent signal afterglow.^21^, ^22^ The presented artifact primarily affected the measurement of uniformity, which measured out of manufacturer’s tolerance. This suggests that QA procedures should either limit CBCT acquisitions done in rapid succession or acquire fewer CBCTs on a monthly basis if this artifact is seen. At the author’s institution, monthly QA protocols were changed from acquiring four CBCT techniques per month to completing the full set of four CBCTs over 2 months. This change in QA protocols has eliminated the prevalence of the artifact.

It was also found that image quality measurements pre‐ and post‐CBCT calibrations were statistically different. As baselines and tolerances are often set using CBCT data, the user could potentially run into an issue where current baselines and tolerances do not appropriately match the measurements that are performed using a new CBCT calibration. To prevent this, the user would need to characterize any changes in image quality metrics and adjust baselines and tolerances accordingly after completing a CBCT calibration.

There has been limited guidance on establishing CBCT QA programs across multiple machines and techniques of the same type. Results from the two‐way ANOVA test (Section 3.A) indicated that CBCT image quality parameters are typically machine‐ and technique‐specific, despite the same machine model, software, and imaging parameters all functioning within manufacturer tolerance. One solution when developing a CBCT monthly QA program would be to define machine‐ and technique‐specific baselines and tolerances. A significant drawback to this approach is the potential change in the expected image quality parameters shown in Section 3.B with calibration of the CBCT system. These changes with calibration would require a new baseline to be established whenever a CBCT technique is recalibrated. Additionally, this route offers no information as to how machines perform relative to one another. Setting individualized machine‐ and technique‐specific baselines introduces the possibility of setting a baseline on an anomalous result, possibly due to equipment performance or differences in QA setup, which then establish an incorrect target value. After looking at the clinical significance of the spread of CBCT data, this work presented a method by which baselines were set for each technique and applied across all machines. It is also important to note that though the spread across all machines of HU values for some HU plugs was greater than the 50 HU Varian tolerance, the spread within each machine and technique was similar to published data.^8^, ^17^, ^18^

Image quality parameters measured in this work are specific to the calculation algorithms used for analysis. For example, SunCHECK Machine™ computes uniformity by looking at the difference in the average HU values across different ROIs. On the other hand, DoseLab Pro (Varian Medical Systems, Palo Alto CA) first adds 1000 to each HU value to convert the HU scale to only positive numbers. Similar ROIs to those used in SNC are defined for evaluating uniformity, but uniformity is computed by using the following formula, where *P_90_* is the 90th percentile of HU values in the ROIs and *P_10_* is the 10th percentile of all HU values in the ROIs:

$$1‐\left(\frac{P_{90}‐P_{10}}{P_{90}+P_{10}}\right)\times 100.$$

Because of the variability in calculation and units for reporting uniformity, the user should understand the algorithm used for calculation of each image quality parameter. This will avoid setting tolerances that may be either too stringent and produce failures that result from measurement variability, or too lenient and not be able to discern degradation in image quality.

The relationship between image quality QA and the incidence of clinically recognizable image quality issues was investigated by Manger et al.^15^ It was found that monthly QA using a Catphan® phantom was not a good predictor of CBCT image quality, mainly due to the reliance on ROI‐based algorithms. Manger et al.^15^ found that automated CBCT analysis is a poor indicator of observable image quality issues performed by human operators. While more work needs to be done to increase the predictive power of CBCT monthly QA, this work serves as a guide for clinics that are starting a comprehensive CBCT image quality program that adheres to current AAPM guidelines.

This work began as a departmental initiative to determine data‐driven baselines and tolerances in order to improve our Linac QA imaging program. Initial results showed variation across all metrics and machines prompting a complete reassessment of the CBCT QA program in order to understand the sources of variability and standardize the process. Differences were found in phantom setup and acquisition (alignment of the phantom with respect to isocenter), phantom used (Catphan 504 vs Catphan 604), the number of phantoms used (each machine used its own Catphan), analysis programs (DoseLab Pro vs SNC Machine), position of analysis ROIs, and test frequency/timing. Staff were retrained, all Catphan 504s were taken out of use, each site used a single Catphan 604, and a single automated CBCT analysis program was installed. The baselines and tolerances determined in this work were only possible after this standardization initiative improved the quality of collected data.

## 5 CONCLUSION

This work focused on the standardization of a CBCT image QA program in an institution with multiple machines and multiple CBCT techniques in clinical use requiring QA. A series of statistically driven studies were completed in order to determine meaningful baselines and tolerances that would be able to identify degradation in image quality while also absorbing routine, expected variability in the measurements. These baselines and tolerances also followed recommendations provided by the AAPM, published in TG‐142 and TG‐179. Two‐way ANOVA tests of CBCT scans from five machines using four imaging techniques showed that, for Varian TrueBeam® machines, CBCT image quality parameters are almost all machine‐ and technique‐dependent indicating that careful consideration is needed when applying institutional‐wide CBCT baselines and tolerances.

During the course of the standardization initiative, two QA scenarios were identified that influence measurement stability: taking multiple CBCTs in rapid succession and performing a CBCT calibration. This work showed that for routine QA protocols that require multiple CBCT acquisitions, the order in which the scans are taken needs to be evaluated. If not, multiple, rapid CBCT acquisitions have the potential to degrade the image quality of QA CBCT scans and can artificially skew QA results into indicating that a machine is out of manufacturer tolerance when it is not. Additionally, this work showed that recalibrating the kV imaging system could change the expected value of image quality parameters. Clinical physicists should be aware of this potential and should consider confirming or re‐establishing image quality baselines after image calibration. Finally, this work presented the baselines and tolerances developed from the institutional standardization initiative. Though the specific values of image quality parameters measured in this work may vary based on the institution, a key conclusion of this work is that the expected value of the parameter can vary: across machines of the same model; with the imaging technique used; on a given machine when the imager is recalibrated; and with the order with which scans are taken. This information can be used as a roadmap at other institutions for what to evaluate when attempting to establish a quantitative CBCT QA program across multiple machines. Furthermore, this work provides a basis for comparison to results measured by other institutions as part of establishing a QA program.

## CONFLICT OF INTEREST

David L. Barbee reports Sun Nuclear Corporation paid for conference travel to speak. None of the other authors has any conflict of interest to disclose.

## Supporting information


**Table S1**. Detailed statistical results for two‐way ANOVA test used to evaluate if image quality parameters are machine or technique dependent. For each main effect (machine, technique) and for the interaction term between the two factors the following are reported: test statistic (*F)*, degrees of freedom (*df)*, and *P*‐value (*p)*.
**Table S2**. Detailed statistical results for unpaired Student's *T*‐test used to evaluate difference in mean between the pre‐calibration and the post‐calibration data for the **head** cone‐beam computed tomography technique. Test statistic (*t*), degrees of freedom (*df*) and *P*‐value (*p*) are reported.
**Table S3**. Detailed statistical results for unpaired Student's *T*‐test used to evaluate difference in mean between the pre‐calibration and the post‐calibration data for the **spotlight** cone‐beam computed tomography technique. *t* is the test statistic and df is the degrees of freedom.
**Table S4**. Detailed statistical results for unpaired Student's *T*‐test used to evaluate difference in mean between the pre‐calibration and the post‐calibration data for the **thorax** cone‐beam computed tomography technique. *t* is the test statistic and df is the degrees of freedom.
**Table S5**. Detailed statistical results for unpaired Student's *T*‐test used to evaluate difference in mean between the pre‐calibration and the post‐calibration data for the pelvis cone‐beam computed tomography technique. *T* is the test statistic and df is the degrees of freedom.
**Table S6**. Institutional baselines and tolerances information for each image quality parameter.
