# Supplementary material for: CBCT image quality QA: Establishing a quantitative program
Source: J Appl Clin Med Phys. 2020 Oct 19;21(11):215–25. doi: 10.1002/acm2.13062 (PMC7701111; doi:10.1002/acm2.13062)
Supplement: Supplementary file 1 — Table S1. Detailed statistical results for two‐way ANOVA test used to evaluate if image quality parameters are machine or technique dependent. For each main effect (machine, technique) and for the interaction term between the two factors the following are reported: test statistic (F), degrees of freedom (df), and P‐value (p). Table S2. Detailed statistical results for unpaired Student's T‐test used to evaluate difference in mean between the pre‐calibration and the post‐calibration data for the head cone‐beam computed tomography technique. Test statistic (t), degrees of freedom (df) and P‐value (p) are reported. Table S3. Detailed statistical results for unpaired Student's T‐test used to evaluate difference in mean between the pre‐calibration and the post‐calibration data for the spotlight cone‐beam computed tomography technique. t is the test statistic and df is the degrees of freedom. Table S4. Detailed statistical results for unpaired Student's T‐test used to evaluate difference in mean between the pre‐calibration and the post‐calibration data for the thorax cone‐beam computed tomography technique. t is the test statistic and df is the degrees of freedom. Table S5. Detailed statistical results for unpaired Student's T‐test used to evaluate difference in mean between the pre‐calibration and the post‐calibration data for the pelvis cone‐beam computed tomography technique. T is the test statistic and df is the degrees of freedom. Table S6. Institutional baselines and tolerances information for each image quality parameter. [file ACM2-21-215-s001.pdf]

Supplemental Material

Table S1: Detailed statistical results for two-way ANOVA test used to evaluate if image quality parameters are machine or technique dependent. For each main effect (machine, technique) and for the interaction term between the two factors the following are reported: test statistic (*F)*, degrees of freedom (*df)*, and p-value (*p)*.

	Machine			Technique			Interaction		
Parameter	*F*	*df*	*p*	*F*	*df*	*p*	*F*	*df*	*p*
Geometric distortion (mm)	141.446	4	<0.001	0.717	3	0.543	8.966	12	<0.001
Spatial resolution (lp/mm)	2.595	4	0.038	137.998	3	<0.001	2.632	12	0.003
Uniformity (HU)	8.216	4	<0.001	19.924	3	<0.001	1.660	12	0.079
Contrast (-)	17.397	4	<0.001	27.478	3	<0.001	9.027	12	<0.001
Noise (HU)	3.755	4	0.006	1796.776	3	<0.001	1.503	12	0.127
Air HU constancy (HU)	4.094	4	0.003	10.937	3	<0.001	4.261	12	<0.001
Teflon HU constancy (HU)	58.301	4	<0.001	216.142	3	<0.001	10.762	12	<0.001
Delrin HU constancy (HU)	29.477	4	<0.001	31.692	3	<0.001	5.322	12	<0.001
Acrylic HU constancy (HU)	26.707	4	<0.001	18.408	3	<0.001	9.773	12	<0.001
Polystyrene HU constancy (HU)	135.380	4	<0.001	414.496	3	<0.001	71.547	12	<0.001
LDPE HU constancy (HU)	126.320	4	<0.001	878.523	3	<0.001	26.850	12	<0.001
PMP HU constancy (HU)	39.225	4	<0.001	1010.181	3	<0.001	141.307	12	<0.001
20 % Bone HU constancy (HU)	55.079	4	<0.001	416.770	3	<0.001	9.690	12	<0.001
50% Bone HU constancy (HU)	164.679	4	<0.001	876.408	3	<0.001	12.014	12	<0.001
Slice thickness (mm)	1.011	4	0.403	169.376	3	<0.001	1.347	12	0.196

Table S2: Detailed statistical results for unpaired Student's T-test used to evaluate difference in mean between the pre-calibration and the post-calibration data for the **head** CBCT technique. Test statistic (*t*), degrees of freedom (*df*) and p-value (*p*) are reported.

	TB1			TB2			TB3		
Parameter	*t*	*df*	*p*	*t*	*df*	*p*	*t*	*df*	*p*
Geometric distortion (mm)	-1.426	12	0.179	0.255	16	0.802	-0.909	16	0.377
Spatial resolution (lp/mm)	0.369	12	0.718	0.889	16	0.387	1.012	16	0.327
Uniformity (HU)	3.239	12	0.007	-2.895	16	0.011	-0.862	16	0.402
Contrast (-)	-3.493	12	0.004	2.000	16	0.063	0.537	16	0.599
Noise (HU)	1.353	12	0.201	-3.038	16	0.008	-0.774	16	0.450
Air HU constancy (HU)	-6.572	12	<0.001	1.428	16	0.172	-0.939	16	0.362
Teflon HU constancy (HU)	6.403	12	<0.001	-1.741	16	0.101	0.091	16	0.929
Delrin HU constancy (HU)	5.857	12	<0.001	-1.805	16	0.090	0.187	16	0.854
Acrylic HU constancy (HU)	5.608	12	<0.001	-1.763	16	0.097	-0.029	16	0.977
Polystyrene HU constancy (HU)	4.464	12	0.001	-2.227	16	0.041	-0.242	16	0.812
LDPE HU constancy (HU)	4.569	12	0.001	-2.023	16	0.060	-1.226	16	0.238
PMP HU constancy (HU)	2.155	12	0.052	-2.298	16	0.035	-0.588	16	0.565
20 % Bone HU constancy (HU)	5.438	12	<0.001	-1.825	16	0.087	0.885	16	0.389
50% Bone HU constancy (HU)	6.286	12	<0.001	-1.576	16	0.135	-1.575	16	0.135
Slice thickness (mm)	2.878	12	0.014	0.492	16	0.630	0.247	16	0.808

Table S3: Detailed statistical results for unpaired Student's T-test used to evaluate difference in mean between the pre-calibration and the post-calibration data for the **spotlight** CBCT technique. t is the test statistic and df is the degrees of freedom.

	TB1			TB2			TB3		
Parameter	t	df	p-value	t	df	p-value	t	df	p-value
Geometric distortion (mm)	-0.055	11	0.957	0.504	15	0.622	-1.325	13	0.208
Spatial resolution (lp/mm)	-0.205	11	0.841	2.152	15	0.048	1.437	13	0.174
Uniformity (HU)	-2.348	11	0.039	-2.974	15	0.009	-3.064	13	0.009
Contrast (-)	-5.442	11	<0.001	1.420	15	0.176	0.806	13	0.435
Noise (HU)	0.015	11	0.988	-0.184	15	0.857	-1.180	13	0.259
Air HU constancy (HU)	2.452	11	0.032	2.938	15	0.010	0.269	13	0.792
Teflon HU constancy (HU)	2.199	11	0.050	-2.671	15	0.017	-2.583	13	0.023
Delrin HU constancy (HU)	1.928	11	0.080	-2.550	15	0.022	-4.126	13	0.001
Acrylic HU constancy (HU)	1.377	11	0.196	-2.545	15	0.022	-4.304	13	0.001
Polystyrene HU constancy (HU)	1.190	11	0.259	-1.884	15	0.079	-5.191	13	<0.001
LDPE HU constancy (HU)	1.398	11	0.190	-3.324	15	0.005	-4.737	13	<0.001
PMP HU constancy (HU)	1.372	11	0.198	-1.536	15	0.145	-5.663	13	<0.001
20 % Bone HU constancy (HU)	0.591	11	0.566	-2.894	15	0.011	-2.637	13	0.021
50% Bone HU constancy (HU)	0.637	11	0.537	-3.102	15	0.007	-3.545	13	0.004
Slice thickness (mm)	1.734	11	0.111	0.985	15	0.340	-1.417	13	0.180

Table S4: Detailed statistical results for unpaired Student's T-test used to evaluate difference in mean between the pre-calibration and the post-calibration data for the **thorax** CBCT technique. t is the test statistic and df is the degrees of freedom.

	TB1			TB2			TB3		
Parameter	t	df	p-value	t	df	p-value	t	df	p-value
Geometric distortion (mm)	1.951	10	0.080	-0.303	13	0.766	-2.893	14	0.012
Spatial resolution (lp/mm)	-2.301	10	0.044	0.168	13	0.869	0.396	14	0.698
Uniformity (HU)	-0.944	10	0.368	-2.492	13	0.027	0.130	14	0.899
Contrast (-)	-2.019	10	0.071	0.985	13	0.343	-1.067	14	0.304
Noise (HU)	2.412	10	0.037	0.403	13	0.694	0.365	14	0.721
Air HU constancy (HU)	-2.920	10	0.015	2.815	13	0.015	0.912	14	0.377
Teflon HU constancy (HU)	4.777	10	0.001	-1.371	13	0.193	-0.074	14	0.942
Delrin HU constancy (HU)	5.909	10	<0.001	-1.112	13	0.286	1.131	14	0.277
Acrylic HU constancy (HU)	5.763	10	<0.001	-1.223	13	0.243	0.630	14	0.539
Polystyrene HU constancy (HU)	3.336	10	0.008	-0.839	13	0.416	0.280	14	0.784
LDPE HU constancy (HU)	2.921	10	0.015	-1.201	13	0.251	1.752	14	0.102
PMP HU constancy (HU)	4.492	10	0.001	-0.688	13	0.503	2.445	14	0.028
20 % Bone HU constancy (HU)	5.087	10	<0.001	-1.623	13	0.129	1.750	14	0.102
50% Bone HU constancy (HU)	4.835	10	0.001	-1.126	13	0.281	-1.007	14	0.331
Slice thickness (mm)	-1.030	10	0.327	0.757	13	0.463	1.143	14	0.272

Table S5: Detailed statistical results for unpaired Student's T-test used to evaluate difference in mean between the pre-calibration and the post-calibration data for the pelvis CBCT technique. T is the test statistic and df is the degrees of freedom.

	TB1			TB2			TB3		
Parameter	t	df	p-value	t	df	p-value	t	df	p-value
Geometric distortion (mm)	0.538	11	0.601	-0.792	14	0.442	-1.930	16	0.072
Spatial resolution (lp/mm)	-2.124	11	0.057	1.415	14	0.179	0.775	16	0.450
Uniformity (HU)	-2.073	11	0.062	-2.717	14	0.017	1.071	16	0.300
Contrast (-)	-1.301	11	0.220	1.235	14	0.237	-0.135	16	0.894
Noise (HU)	-1.013	11	0.333	0.341	14	0.738	-1.327	16	0.203
Air HU constancy (HU)	-1.572	11	0.144	2.217	14	0.044	0.502	16	0.623
Teflon HU constancy (HU)	3.718	11	0.003	-1.768	14	0.099	-1.442	16	0.169
Delrin HU constancy (HU)	2.396	11	0.035	-0.777	14	0.450	-0.594	16	0.561
Acrylic HU constancy (HU)	2.114	11	0.058	-0.632	14	0.538	-0.743	16	0.468
Polystyrene HU constancy (HU)	2.089	11	0.061	-1.105	14	0.288	-0.685	16	0.503
LDPE HU constancy (HU)	1.992	11	0.072	-0.853	14	0.408	-0.782	16	0.446
PMP HU constancy (HU)	1.707	11	0.116	0.312	14	0.759	0.203	16	0.842
20 % Bone HU constancy (HU)	3.067	11	0.011	-1.354	14	0.197	-0.579	16	0.571
50% Bone HU constancy (HU)	2.648	11	0.023	-1.402	14	0.183	-2.106	16	0.051
Slice thickness (mm)	-0.124	11	0.904	-0.310	14	0.761	0.545	16	0.593

Table S6: Institutional baselines and tolerances information for each image quality parameter

Parameter	Baseline	Spotlight	Pelvis	Head	Thorax	Tolerance
Geometric distortion (mm)	0	0	0	0	0	±1
Spatial resolution (lp/mm)	Mean	0.35	0.32	0.42	0.32	±2σ
Uniformity (HU)	0	0	0	0	0	±40
Contrast (-)	Mean	0.93	0.93	0.94	0.92	±2σ
Noise (HU)	Mean	5.77	6.08	23.30	11.39	±2σ
Air HU Constancy (HU)	Mean	-999.10	-999.36	-997.72	-999.36	±50
Teflon HU Constancy (HU)	Mean	981.06	978.61	1047.92	1000.70	±2σ/±50 HU
Delrin HU Constancy (HU)	Mean	368.76	367.42	384.63	378.47	±50
Acrylic HU Constancy (HU)	Mean	129.85	126.58	133.10	132.58	±50
Polystyrene HU Constancy (HU)	Mean	-29.60	-32.79	-35.54	-29.54	±50
LDPE HU Constancy (HU)	Mean	-88.75	-91.63	-98.26	-90.26	±50
PMP HU Constancy (HU)	Mean	-181.76	-185.17	-192.38	-184.87	±50
20% Bone HU Constancy (HU)	Mean	254.15	254.76	295.07	266.11	±50
50% Bone HU Constancy (HU)	Mean	744.80	751.50	868.15	774.39	±2σ/±50 HU
Slice thickness (mm)	Mean	1.89	2.12	1.85	2.13	±2σ
